# Activity and Damage Indices for Sjögren’s Disease

**DOI:** 10.3390/jcm15041478

**Published:** 2026-02-13

**Authors:** Elodie Portier, Maxime Beydon, Simon J. Bowman, Raphaèle Seror

**Affiliations:** 1 Department of Immuno-Rheumatology, Hôpital Bicêtre, Assistance Publique—Hôpitaux de Paris, Université Paris-Saclay, 94270 Le Kremlin-Bicêtre, France; elodie.portier@aphp.fr; 2 INSERM, Institut Pierre Louis d’Epidémiologie et de Santé Publique, Equipe PEPITES, AP-HP, Hôpital Pitié Salpêtrière, Département de Santé Publique, Centre de Pharmacoépidémiologie (Cephepi), Sorbonne Université, 75013 Paris, France; maxime.beydon@aphp.fr; 3 Rheumatology Department, University Hospitals Birmingham National Health Service Foundation Trust, Birmingham B15 2TH, UK; simon.bowman@uhb.nhs.uk; 4 Centre de Recherche en Epidémiologie et Santé des Populations, Université Paris-Saclay, Université Versailles Saint Quentin, Inserm, Institut Gustave Roussy, 94805 Villejuif, France

**Keywords:** Sjögren’s disease, disease activity, damage

## Abstract

Primary Sjögren’s disease (SjD) is a systemic autoimmune disease with heterogeneous clinical manifestations, ranging from isolated glandular dysfunction to multisystem involvement and severe complications. This variability complicates disease management, particularly the assessment of disease activity and long-term outcomes. Disease activity in SjD encompasses both systemic manifestations and patient-reported outcomes (PROs). The EULAR Sjögren’s Syndrome Disease Activity Index (ESSDAI) remains the standard for systemic activity, while the ESSPRI complements these scores by capturing subjective symptom burden. These tools have limitations because they do not evaluate the same manifestations of SjD and may therefore respond differently to treatment. Thus, composite outcomes such as the Sjögren’s Tool for Assessing Response (STAR) and the Composite of Relevant Endpoints for Sjögren Syndrome (CRESS) have been developed. They integrate improvements in ClinESSDAI, PROs, biological parameters and objective dryness, providing a more comprehensive measure of disease activity. Assessment of damage, defined as irreversible sequelae, remains an unmet need in SjD. Damage is a reflection of the natural evolution of the disease, and is also relevant to measuring the impact and health economic effects of therapeutic interventions. Existing measures, including cumulative ESSDAI, track longitudinal activity and reflect the severity of the disease, but do not specifically capture damage. In summary; SjD assessment requires complementary tools for systemic activity and PROs, with composite scores a recent innovation. An important remaining gap is a validated method to evaluate damage, essential to fully evaluate disease progression.

## 1. Introduction and Methods

Primary Sjögren’s disease (SjD) encompasses a broad clinical spectrum, from isolated glandular dysfunction to multisystem inflammatory disease and severe complications including lymphoma [1,2]. This variability influences almost every aspect of disease management, including the assessment of response to treatment in its various components (disease activity, patient-reported outcomes). Accurate measurement of these parameters is essential both for identifying potential disease modifying drugs (DMARDs), and to assess disease activity changes over time.

For diseases with a smaller number of dimensions to consider such as rheumatoid arthritis, designing activity scores is arguably intuitive. In SjD, considering the wide variety of symptoms and the now increasingly recognized clusters of disease expression [3], designing one index that embraces all possible disease activity manifestations and another to capture all the relevant aspects of damage is challenging. This is necessary, however, to allow evaluation of treatment efficacy and to conduct clinical and translational research.

Furthermore, the heterogeneity of the disease is also reflected in the assessment of its various manifestations, since it includes both objective measures of systemic activity, patient-reported outcomes (PROs) and objective glandular involvement.

One of the most widely used scores to evaluate systemic activity in SjD is the EULAR Sjögren’s Syndrome Disease Activity Index (ESSDAI) [4]. It was first published in 2010 and still is a core reference in assessing disease activity in SjD [5], as is its derived version, the clinical ESSDAI (clinESSDAI) [6]. Nevertheless, although designed to measure disease activity, ESSDAI does not correlate with aspects of the disease captured by PROs [7,8,9]. For this reason, the complementary measure the EULAR Sjögren Syndrome Patient Reported Index (ESSPRI) has been developed in parallel with the ESSDAI [7,10].

The ESSDAI is an activity score, designed to assess organ involvement where recovery due to active disease is still reversible. It was designed to exclude evaluation of organ damage, so as to be able to detect improvement in evaluation of treatment efficacy [11]. To date, there is no validated definition of damage, and there are a very limited set of scoring systems proposed to assess organ damage in SjD [12,13]; this needs to be addressed.

Therefore, in this review, we will critically examine the current state of knowledge about outcome measures used to assess disease activity and response to treatment overall in SjD, explore opportunities for developing tools to evaluate organ damage, and discuss why these assessment tools are essential.

For this work, we performed a comprehensive literature research on Pubmed and included recent unpublished works (recent meeting abstracts). We then selected all relevant works and previous reviews to summarize the key messages in this review article.

## 2. Background: Development of Disease Activity Scores in SjD

Disease activity in SjD can broadly be distinguished in two broad categories: PROs including sicca symptoms (dryness, pain and fatigue) and systemic activity (organ involvement that is reversible).

The easiest way of measuring fatigue and pain is to use 0–10 visual analog scales (or Likert scoring equivalents), rating from “no fatigue (pain)” to “worst fatigue (pain)”. Another option to assess fatigue is to use a questionnaire addressing different components of fatigue. Questionnaires can be “unidimensional” such as the Functional Assessment of Chronic Illness Therapy-Fatigue (FACIT-F) scale [14]; they also can be “multidimensional” with the evaluation of different aspects of fatigue (e.g., severity, physical and mental fatigue, impact on daily activities…) such as the Multidimensional Fatigue Inventory [15]. A large number of questionnaire tools assessing fatigue exist and can be used in all relevant diseases [16]. The sicca symptoms of SjD have been evaluated in clinical trials through various scales, mostly using visual analog scales [17,18,19,20], as well as specific scales for fatigue [21].

The Profile of Fatigue and Discomfort-Sicca Symptoms Inventory (PROFAD-SSI) questionnaire was specifically developed with qualitative data from interviews of patients with SjD, and, subsequently, a short from of the PROFAD-SSI was proposed [22]. The brief form of the PROFAD-SSI, alongside the empiric measurement of sicca symptoms, pain and fatigue, inspired the shortened practical score, the ESSPRI [10]. The ESSPRI correlates well with the more complex dimensions captured by the comprehensive assessment score PROFAD-SSI, and the patient global assessment which served as a gold standard for its development even in an independent population [7] (Table 1). Moreover, de Wolff et al. confirmed, as initially demonstrated in the development of ESSPRI, that separate dryness assessments only add little value to the overall dryness question of the ESSPRI [23].

The ESSPRI assigns equal weight to its three domains (dryness, fatigue and pain) and calculates an overall score by averaging their respective visual numerical scales. Each scale reflects symptom intensity over the preceding two weeks and ranges from 0 to 10. A score of ≥5 is used to define a high level of symptoms and thus the cut-off of <5 is used for a patient acceptable symptom state (PASS) [24,25]. Minimal clinically important improvement requires a one point or 15% reduction compared with baseline.

The Sjögren’s Syndrome Disease Activity Index (SSDAI) [12] and the Sjögren’s Systemic Clinical Activity Index (SCAI) [26] were the first tools to assess disease activity, which paved the way for the evaluation of various domains with relative weights according to physicians’ appreciation of severity.

These efforts were later pursued and led to the validation of the ESSDAI [5], which is now well known and recognized as the gold standard by the SjD community. It has been used in every modern SjD clinical trial [27,28,29,30]. The development of the ESSDAI was based on an international collaboration of experts, with several steps including [5]:

- Identification of organ-specific “domains” contributing to disease activity which were classified in 3 or 4 levels according to their severity
- Generation of realistic vignettes of patients with SjD for which all possible systemic complications were represented.
- Use of vignettes and patient profiles to calculate the 0–10 physician global assessment (PGA) scale
- Determination of domain weights in ESSDAI by assessing correlation between disease activity (assessed by the PGA scale) and domains.

The ESSDAI quantifies disease activity using 12 domains (Table 2). By design, it does not assess PROs that are known to be influenced by non SjD related symptoms and comorbidities. In other words, the ESSDAI is specifically intended to measure disease activity independently of PROs, which were already known not to correlate well with systemic disease activity in target organs. An ESSDAI score of 1 to 4 corresponds to mild disease activity, 5 to 13 to moderate, and ≥14 to high. Improvement of at least 3 points usually defines a treatment response (minimal clinically important difference).

The ClinESSDAI was derived from the ESSDAI to evaluate disease activity independently of biological parameters (i.e., B-cell biomarkers), to prevent score variations driven solely by the drug’s biological effects, and to limit correlated variables in experimental studies evaluating biomarkers.

## 3. Assessing Disease Manifestations and Activity in SjD: Strengths and Limits of the ESSPRI and ESSDAI

It should be recalled that the ESSDAI and ESSPRI were developed jointly, and designed to be complementary as it was already known that there was no direct correlation between systemic activity and PROs [9,31].

After the development of these scores and adoption by the scientific community, they have contributed to positive changes in the management of the disease. The ESSDAI has been widely accepted as the main tool to assess disease activity. Clinical trials evaluating patients with SjD have confirmed its sensitivity to change and, for the first time, some trials have delivered positive primary outcomes [27,29,30,32], including phase 3 trials [33,34], in contrast to other previous unvalidated measures of disease activity when they were used as primary endpoints. The ESSDAI was also used and validated in large cohorts of patients with SjD [35,36], because of its clinical relevance, as the ESSDAI is associated both with disease activity and with long term outcomes. For example, the ESSDAI is associated with the risk of lymphoma and death [37,38]. An ESSDAI disease activity cut off has been shown to be clinically meaningful as patients who maintained an ESSDAI low disease activity state had better long term outcomes [39].

The ESSPRI has demonstrated greater reliability but a lower sensitivity to change than the ESSDAI [8]. However, the main limitation of PROs lies in the subjective and non-specific nature of the domains assessed. Fatigue or pain may be influenced by various comorbidities and co-conditions, such as fibromyalgia, anxiety and depression. In addition, symptoms such as dryness and pain may reflect both irreversible tissue damage and active inflammatory disease. Consequently, when PROs such as the ESSPRI were used as the sole assessment tools—as was done in earlier trials [18,20]—treatment response may be estimated incorrectly. Overall, multiple factors contribute to PRO scores, which may capture disease activity, established damage, or symptoms that are not directly related to SjD itself.

In addition, it has been clearly confirmed in cluster studies that systemic activity does not necessarily correlate with the subjective symptom burden [3,40], with possible distinct molecular activation pathways according to disease phenotypes [41]. Moreover, patients may present with a high level of symptoms (as assessed through PROs), which can be associated with either high or low levels of systemic disease activity. To account for this discrepancy, some trials have opted to include two cohorts: the first with moderate to severe disease activity according to the ESSDAI, and the second cohort without, or with low systemic activity, but with a clinically significant symptom burden according to the ESSPRI [27,29]. Nevertheless, this approach does not fully address the challenge that, in patients with high systemic disease activity, the primary endpoint of the ESSDAI may be reached without improving any PRO, and thus without relieving the patient’s symptom burden. In order to resolve this issue, a proposed solution is to combine these outcomes in a single composite outcome.

## 4. A Better Option for Disease Activity Assessment: Composite Outcomes

As a reminder, taken individually, the ESSDAI and ESSPRI assess only part of the overall SjD features. To obtain a comprehensive and clinically relevant view of the treatment response, it is necessary to integrate multiple dimensions through composite scores.

Two similar composite outcomes have been designed in order to address these issues: the Sjögren’s Tool for Assessing Response (STAR) and the Composite of Relevant Endpoints for Sjögren Syndrome (CRESS) (Table 3) [42,43]. In these composite outcomes, improvement in disease activity through clinESSDAI needs to be accompanied by improvement in either ESSPRI or objective dryness. The STAR and CRESS include five domains (systemic activity, sicca symptoms, salivary gland function, lachrymal gland function, and biologic parameters).

The main differences between these composite endpoints are twofold. Firstly, they differ in domain weighting: STAR defines two major domains and requires improvement in at least one of them, whereas CRESS assigns equal weight to each domain. Secondly, they differ in the definition of clinESSDAI response: in STAR, the response is defined as a ≥3-point reduction, while in CRESS it is defined as achieving a clinESSDAI score of <5.

In an initial validation step, after discussion among 78 experts and 20 patients, STAR candidates were tested against nine clinical trials. Overall positivity was reassessed by experts rather than being interpretated solely based on their variable primary outcomes [43]. The final candidate was chosen based on its ability to best discriminate negative from positive trials, and clinical relevance. CRESS was also validated in 3 independent trials [42]. Moreover, STAR demonstrates its ability to accurately detect treatment efficacy in 16 recent trials [44].

Given its apparent robustness regarding its ability to discriminate treatment effect in clinical trials while correlating with PROs, the European Medical Agency has written a letter of support for STAR [45].

## 5. Role of Other Tools in the Assessment of Disease Activity (Imaging and Histology)

Histopathology of salivary gland biopsies is the principal standard test to diagnose SjD. It is more difficult to assess its relevance for the follow-up of patients with SjD and particularly to assess the precise impact of treatments on target tissue. Until recently, the majority of biological treatments studied in SjD only minimally induce B-cell depletion in biopsies performed during follow-up [46]. However, more recent strategies, such as the combination of Belimumab and Rituximab [47] seem to have a much larger effect on tissue depletion of B-cells, as for example in a trial of Ianalumab [48]. Nevertheless, even if histological changes appear to be an interesting goal, it is likely to be difficult to perform repeated biopsies in patients with SjD, whether from an ethical or organizational standpoint. Moreover, the benefit for patients in improvement from a purely histological perspective, if this is not correlated with clinical improvement is questionable. Nevertheless, assessing the link between tissue B-cell depletion and clinical response can be an important research objective.

Salivary gland ultrasonography (SGUS) can be a useful non-invasive tool, supporting the diagnosis of SjD, with a semi-quantitative scoring system proposed by the International Outcome Measures in Rheumatology (OMERACT) group. It is not included in the ACR/EULAR classification criteria, but its additive role in the diagnosis of SjD has been suggested by several studies [49,50]. SGUS scores correlate in some studies with salivary flow rates [51] and there may also be an association with high systemic disease activity [52]. It is not part of the ESSDAI score but has been included in the composite scores (STAR, CRESS). SGUS may play a role in the assessment of disease activity in clinical trials, with several studies showing improvements in SGUS features [53,54,55], although this is not the case in all studies [56]. SGUS may also have a role in the detection of lymphoma although further research may be needed to determine its sensitivity and specificity in this regard [57]. In summary, SGUS is a useful clinical tool and its precise role in clinical studies and outcome assessment needs to be studied further [55].

## 6. Evaluation of Damage in SjD

In autoimmune diseases, damage refers to irreversible organ sequelae resulting from prior disease activity, concomitant disease or treatment toxicity [58,59]. Usually, attempts to capture damage are not necessarily aimed at determining the precise causation; not least because sometimes it may be impossible to do so. Consider, for example, a female patient with Systemic Lupus Erythematosus (SLE) who develops a cataract. This might be linked to the fact that both her parents had cataracts, the corticosteroids that she has been prescribed or because she has concomitant diabetes. It would be impossible to know if one or two or all three factors are involved.

By design, the ESSDAI score exclusively measures active systemic disease activity, and thus a feature that is stable for ≥12 months is excluded. This time-frame was chosen to align with the typical time-frame of a clinical trial and to avoid a scenario where a decrease of the score does not reflect a true improvement. The precise timepoint when activity ‘morphs’ into damage is hard to ascertain, unless an arbitrary time point is decided. An ESSDAI component that is stable for 12 months or more is, therefore, considered to have ‘converted’ into damage. Effectively, once symptoms become fixed, and no longer reflect active inflammation, the ESSDAI score decreases (as such manifestations are no longer scored). It ensures sensitivity to change of the score. For instance, a patient with acute interstitial pneumonia who fails to respond to treatment has a decrease in ESSDAI after 12 months, even though the condition has merely evolved from active inflammation to chronic sequelae. This avoids inclusion in clinical trials of patients with long-standing manifestations that might not respond to treatment. Similarly, the objective measures of sicca proposed in STAR distinguish between salivary or lachrymal gland dysfunction due to potentially reversible lymphoid infiltration, and dysfunction likely related to irreversible glandular atrophy. Finally, in PROs such as ESSPRI, the drivers of symptoms are multiple (activity, damage, comorbidities, anxiety, fibromyalgia, depression…) and their presence cannot be attributed to a unique component of activity or damage.

However, by design, ESSDAI does not measure damage in SjD, and it should be noted that, to date, there is no validated definition of irreversible organ damage in SjD.

Only two scores have focused on damage: (1) the Sjögren’s Syndrome Disease Damage Index (SSDDI) [12], which was developed in 2007 in twelve Italian centers and (2) the Sjogren’s Syndrome Damage Index (SSDI) developed in the UK [13]. SSDDI is simpler, containing 8 items. SSDI has 29 items including ocular, oral and systemic domains. Their usage has been scarce over the years [13], as most studies focused on acute activity rather than damage.

In order to capture longitudinal activity, we have proposed a cumulative ESSDAI (cumESSDAI) which sums the maximum of each domain activity over time [60,61]. This measure captures both the diversity of domains involved and the accumulation of activity over time, but not necessarily accrual of sequelae. Damage should rather reflect additive, longitudinal assessment of organ impairment. Nevertheless, it is likely that both are correlated.

In SLE, association between activity scores and accrual of damage has been demonstrated, as well as correlations between damage and mortality [62]. These scores allow for secondary analysis of trials focusing specifically on organ damage, which are sometime regarded as a proxy of disease severity [63,64]. In SjD, ESSDAI and extra-glandular activity have been positively associated with mortality [37,65], possibly through organ damage which alters vital functions or by exposing patients to toxic treatments for example in SjD in the case of lymphoma. In SLE, the damage score the Systemic Lupus International Combined Clinics/American College of Rheumatology Damage Index (SLICC/ACR) in SLE [62], incorporates irreversible treatment toxicity as part of damage which might better integrate disease burden, but also may cause some confusion in interpretation.

SLE is also an exemplar, relevant to other multi-dimension diseases such as SjD, of how regulatory authorities have assessed the economic impact of novel therapy by evaluating damage prevention to justify the cost of the therapy, e.g., of Belimumab [66].

## 7. Future Research Directions

It is important to note that the STAR currently remains a candidate score. Further studies will be conducted to determine the optimal improvement threshold for each of its components (ESSDAI, ESSPRI). It may be suggested that a STAR definition including a 5-point decrease in the ESSDAI represents a particularly attractive alternative. In addition, a more stringent threshold for the ESSPRI (≥2 instead of 1) could also be considered. Indeed, several ESSPRI thresholds were tested in the phase 2 study evaluating the efficacy of Ianalumab [67]. These different options have been tested in independent recent trials [44], and will be further tested in the NECESSITY trial. Nevertheless, it should be noted that no single outcome measure in autoimmune diseases has been evaluated across such a large number of individual clinical trials or studied so extensively prior to final validation. At this stage, it can therefore be reasonably concluded that STAR is a valid outcome measure, able to consistently and uniformly assess treatment response, with good sensitivity to change across the full spectrum of SjD patients, whether they present with predominantly systemic activity or predominantly symptoms.

As it was previously detailed, the lack of a standardized and validated index for damage is the main missing link in the assessment of patients with SjD. In the same way as for SLE, now that better activity indices have emerged, it is time to develop a validated damage score in SjD. Domains to be considered have already been identified in ESSDAI, and now STAR. The objective is to develop a validated damage score for SjD that captures overall, systemic, and glandular irreversible damage. The score should rely on clearly defined and clinically relevant damage domains, be applicable across cohorts and clinical trials, and allow standardized assessment of long-term disease burden. It should demonstrate construct validity through correlations with existing damage-related tools (such as the damage Visual Analog Scale and cumESSDAI) and be suitable for studying associations between damage accumulation and long-term outcomes, including mortality, lymphoma, and severe systemic complications. The goal is to demonstrate that controlling all the manifestations included in the score will prevent organ damage, and ultimately improve long-term outcomes.

## 8. Conclusions

SjD is a widely heterogeneous disease, with various clinical and paraclinical manifestations. This heterogeneity complicates disease management, starting with the selection and use of appropriate outcome measures for the assessment of response to treatment. ESSDAI and ESSPRI have shown their complementarity in the assessment of systemic activity and PROs and they have been increasingly used in therapeutic trials over the years [68].

They have limitations when used separately, mostly overcome by their complementarity, which has led to the development of composite outcomes (CRESS, STAR), which have been gradually adopted since their validation.

The main tool currently lacking from our armamentarium concerns the assessment of damage in SjD. We are now proposing consideration of the cumESSDAI as it reflects the accrual of disease activity, and is therefore likely to be correlated with damage. The development of a validated method to evaluate damage appears essential to fully evaluate disease progression and the impact of therapeutic strategies.

## Figures and Tables

**Table 1:** Composition and calculation of ESSPRI.

Visual Numerical Scale (0–10)	Score
Pain	0–10
Fatigue	0–10
Dryness	0–10
Total score: mean of the 3 sub-scores, 0–10	

**Table 2:** Composition and calculation of ESSDAI.

Domain	Activity Level	Score	Description
**Constitutional** Exclusion of fever of infectious origin and voluntary weight loss	No	0	Absence of the following symptoms
	Low	3	Mild or intermittent fever (37.5–38.5 °C)/night sweats and/or involuntary weight loss of 5 to 10% of body weight
	Moderate	6	Severe fever (>38.5 °C)/night sweats and/or involuntary weight loss of >10% of body weight
**Lymphadenopathy and lymphoma** Exclusion of infection	No	0	Absence of the following features
	Low	4	Lymphadenopathy ≥1 cm in any nodal region or ≥2 cm in inguinal region
	Moderate	8	Lymphadenopathy ≥2 cm in any nodal region or ≥3 cm in inguinal region, and/or splenomegaly (clinically palpable or by imaging)
	High	12	Current malignant B-cell proliferative disorder
**Glandular** Exclusion of infection or stone	No	0	Absence of glandular swelling
	Low	2	Small glandular swelling with enlarged parotid (≤3 cm), or limited submandibular (≤2 cm) or lachrymal swelling (≤1 cm)
	Moderate	4	Major glandular swelling with enlarged parotid (>3 cm), or important submandibular (>2 cm) or lachrymal swelling (>1 cm)
**Articular** Exclusion of osteoarthritis	No	0	Absence of currently active articular involvement
	Low	2	Arthralgias in hands, wrists, ankles and feet accompanied by morning stiffness (>30 min)
	Moderate	4	1 to 5 (of 28 total count) synovitis
	High	6	≥6 (of 28 total count) synovitis
**Cutaneous** Rate as “No activity” stable long-lasting features related to damage	No	0	Absence of currently active cutaneous involvement
	Low	3	Erythema multiforma
	Moderate	6	Limited cutaneous vasculitis, including urticarial vasculitis, or purpura limited to feet and ankle, or subacute cutaneous lupus
	High	9	Diffuse cutaneous vasculitis, including urticarial vasculitis, or diffuse purpura, or ulcers related to vasculitis
**Pulmonary** Rate as “No activity” stable long-lasting features related to damage, or respiratory involvement not related to the disease	No	0	Absence of currently active pulmonary involvement
	Low	5	Persistent cough due to bronchial involvement with no radiographic abnormalities on radiography OR radiological/HRCT evidence of interstitial lung disease with: No breathlessness and normal lung function test.
	Moderate	10	Moderately active pulmonary involvement, such as interstitial lung disease shown by HRCT with shortness of breath on exercise (NHYA II) or abnormal lung function (tests restricted to: 70% > DLCO ≥ 40% or 80% > FVC ≥ 60%)
	High	15	Highly active pulmonary involvement, such as interstitial lung disease shown by HRCT with shortness of breath at rest (NHYA III, IV) or with abnormal lung function tests: DLCO < 40% or FVC < 60%
**Renal** Rate as “No activity” stable long-lasting features related to damage, and renal involvement not related to the disease. If biopsy has been performed, please rate activity based on histological features first	No	0	Absence of currently active renal involvement with proteinuria < 0.5 g/d, no hematuria, no leucocyturia, no acidosis, or long-lasting stable proteinuria due to damage
	Low	5	Evidence of mild active renal involvement, limited to tubular acidosis without renal failure OR glomerular involvement with proteinuria (between 0.5 and 1 g/d) and without hematuria or renal failure (GFR ≥ 60 mL/min)
	Moderate	10	Moderately active renal involvement, such as tubular acidosis with renal failure (GFR < 60 mL/min) OR glomerular involvement with proteinuria between 1 and 1.5 g/d and without hematuria or renal failure (GFR ≥ 60 mL/min) OR histological evidence of extra-membranous glomerulonephritis or important interstitial lymphoid infiltrate
	High	15	Highly active renal involvement, such as glomerular involvement with proteinuria > 1.5 g/d or hematuria or renal failure (GFR < 60 mL/min), OR histological evidence of proliferative glomerulonephritis or cryoglobulinemia related renal involvement
**Muscular** Exclusion of weakness due to corticosteroids	No	0	Absence of currently active muscular involvement
	Low	6	Mild active myositis shown by abnormal EMG, MRI or biopsy with no weakness and CK (N < CK ≤ 2N)
	Moderate	12	Moderately active myositis proven by abnormal EMG, MRI or biopsy with weakness (maximal deficit of 4/5), or elevated CK (2N < CK ≤ 4N),
	High	18	Highly active myositis shown by abnormal EMG, MRI or biopsy with weakness (deficit ≤ 3/5) or elevated CK (>4N)
**PNS** Rate as “No activity” stable long-lasting features related to damage or PNS involvement not related to the disease	No	0	Absence of currently active PNS involvement
	Low	5	Mild active peripheral nervous system involvement, such as pure sensory axonal polyneuropathy shown by NCS or trigeminal (V) neuralgia or proven small fibre neuropathy
	Moderate	10	Moderately active peripheral nervous system involvement shown by NCS, such as axonal sensory-motor neuropathy with maximal motor deficit of 4/5, pure sensory neuropathy with presence of cryoglobulinemic vasculitis, ganglionopathy with symptoms restricted to mild/moderate ataxia, inflammatory demyelinating polyneuropathy (CIDP) with mild functional impairment (maximal motor deficit of 4/5 or mild ataxia), OR cranial nerve involvement of peripheral origin (except trigeminal (V) neralgia)
	High	15	Highly active PNS involvement shown by NCS, such as axonal sensory-motor neuropathy with motor deficit ≤ 3/5, peripheral nerve involvement due to vasculitis (mononeuritis multiplex etc.), severe ataxia due to ganglionopathy, inflammatory demyelinating polyneuropathy (CIDP) with severe functional impairment: motor deficit ≤ 3/5 or severe ataxia
**CNS** Rate as “No activity” stable long-lasting features related to damage or CNS involvement not related to the disease	No	0	Absence of currently active CNS involvement
	Moderate	10	Moderately active CNS features, such as cranial nerve involvement of central origin, optic neuritis or multiple sclerosis-like syndrome with symptoms restricted to pure sensory impairment or proven cognitive impairment
	High	15	Highly active CNS features, such as cerebral vasculitis with cerebrovascular accident or transient ischemic attack, seizures, transverse myelitis, lymphocytic meningitis, multiple sclerosis-like syndrome with motor deficit.
**Hematological** For anemia, neutropenia, and thrombopenia, only auto-immune cytopenia must be considered. Exclusion of vitamin or iron deficiency, drug-induced cytopenia	No	0	Absence of auto-immune cytopenia
	Low	2	Cytopenia of auto-immune origin with neutropenia (1000 < neutrophils < 1500/mm^3^), and/or anemia (10 < hemoglobin < 12 g/dL), and/or thrombocytopenia (100,000 < platelets < 150,000/mm^3^) OR lymphopenia (500 < lymphocytes < 1000/mm^3^)
	Moderate	4	Cytopenia of auto-immune origin with neutropenia (500 ≤ neutrophils ≤ 1000/mm^3^), and/or anemia (8 ≤ hemoglobin ≤ 10 g/dL), and/or thrombocytopenia (50,000 ≤ platelets ≤ 100,000/mm^3^) OR lymphopenia (≤500/mm^3^)
	High	6	Cytopenia of auto-immune origin with neutropenia (neutrophils < 500/mm^3^), and/or or anemia (hemoglobin < 8 g/dL) and/or thrombocytopenia (platelets < 50,000/mm^3^)
**Biological**	No	0	Absence of any of the following biological feature
	Low	1	Clonal component and/or hypocomplementemia (low C4 or C3 or CH50) and/or hypergammaglobulinemia or high IgG level between 16 and 20 g/L
	Moderate	2	Presence of cryoglobulinemia and/or hypergammaglobulinemia/high IgG level > 20 g/L, and/or recent onset hypogammaglobulinemia or recent decrease of IgG level < 5 g/L

**Table 3:** Composition and calculation of CRESS and STAR.

Domain	Definition of Response	STAR (Points)	CRESS (Points)
Systemic activity (STAR)	Decrease of ClinESSDAI ≥ 3	3	
Systemic activity (CRESS)	ClinESSDAI score < 5		3
Patient-reported outcome	Decrease of ESSPRI ≥ 1 point or ≥15%	3	1
Lachrymal gland function (mean of both eyes)	Schirmer I: If abnormal score at baseline: increase ≥ 5 mm from baseline OR Ocular Staining Score: If abnormal score at baseline: decrease ≥ 2 points from baseline OR If both normal at baseline: no change to abnormal	1	1
Salivary gland function	Unstimulated Whole Salivary Flow (UWSF): If score > 0 at baseline: increase ≥ 25% from baseline If score = 0 at baseline: any increase in UWSF OR Ultrasound: Decrease ≥ 25% in total Hocevar score	1	1
Biological	IgG decrease ≥ 10% OR Rheumatoid factor decrease ≥ 25% (total RF or RF-IgM (IU/mL))	1	1
Responder definition		≥5 points	≥3 points

## Data Availability

No data were created for this review.

## Abbreviations

The following abbreviations are used in this manuscript:

ACR	American College of Rheumatology
CIDP	Chronic Inflammatory Demyelinating Polyneuropathy
CNS	Central Nervous System
CRESS	Composite of Relevant Endpoints for Sjögren Syndrome
CK	Creatine Kinase
DLCO	Diffusing Capacity of the Lung for Carbon Monoxide
DMARDs	Disease Modifying Anti-Rheumatic Drugs
EMG	Electromyogram
ESSDAI	EULAR Sjögren’s Syndrome Disease Activity Index
ESSPRI	EULAR Sjögren’s Syndrome Patient Reported Index
FACIT-F	Functional Assessment of Chronic Illness Therapy – Fatigue
FVC	Forced Vital Capacity
GFR	Glomerular Filtration Rate
HRCT	High-Resolution Computed Tomography
IgG	Immunoglobulin G
MRI	Magnetic Resonance Imaging
NCS	Nerve Conduction Studies
NYHA	New York Heart Association
PASS	Patient acceptable symptom state
PGA	Physician Global Assessment
PNS	Peripheral Nervous System
PROs	Patient-Reported Outcomes
PROFAD-SSI	Profile of Fatigue and Discomfort – Sicca Symptoms Inventory
RF	Rheumatoid Factor
SCAI	Sjögren’s Systemic Clinical Activity Index
SjD	Sjögren’s Disease
SLE	Systemic Lupus Erythematosus
SSDAI	Sjögren’s Syndrome Disease Activity Index
SSDDI	Sjögren’s Syndrome Disease Damage Index
SSDI	Sjögren’s Syndrome Damage Index
SGUS	Salivary gland ultrasonography
STAR	Sjögren’s Tool for Assessing Response
UWSF	Unstimulated Whole Salivary Flow
